# Practical implications of ICD-11 personality disorder classifications

**DOI:** 10.1186/s12888-024-05640-3

**Published:** 2024-03-07

**Authors:** Bing Pan, Wei Wang

**Affiliations:** 1 Department of Psychiatry, Second Affiliated Hospital, Zhejiang University School of Medicine, Zhejiang University, Hangzhou, China; 2 Department of Psychology, Norwegian University of Science and Technology, Trondheim, Norway

**Keywords:** Dimensional diagnosis, DSM-5, ICD-11, Personality disorder assessment, Personality disorder in adolescence

## Abstract

Personality disorders (PDs) are associated with an inferior quality of life, poor health, and premature mortality, leading to heavy clinical, familial, and societal burdens. The International Classification of Diseases-11 (ICD-11) makes a thorough, dramatic paradigm shift from the categorical to dimensional diagnosis of PD and expands the application into adolescence. We have reviewed the recent literature on practical implications, and severity and trait measures of ICD-11 defined PDs, by comparing with the alternative model of personality disorders in the fifth edition of the Diagnostic and Statistical Manual of Mental Disorders (DSM-5), by mentioning the relevance in forensic and social concerns, and by referencing the developmental implication of life span, especially in adolescence. Study results strongly support the dimensional utility of ICD-11 PD diagnosis and application in adolescence which warrants early detection and intervention. More evidence-based research is needed along the ICD-11 PD application, such as its social relevance, measurement simplification, and longitudinal design of lifespan observation and treatment.

## Introduction

Personality disorders (PDs) are associated with several areas of human daily functioning, such as affectivity, impulse control, perception, thinking patterns, and reaction to stress factors [1]. These disorders impose noticeable clinical, familial, and societal burdens [2, 3]. Moreover, PDs have high comorbidity with other mental disorders, influencing outcomes [4, 5], and they increase the treatment difficulties of chronic psychosomatic disorders [6]. The life expectancy for patients with PDs and comorbid depression is at least 1.5 years shorter for men and 1.6 years shorter for women as compared to patients with depression only [7].

In general population, PDs are prevalent as high as 12.16% in Western countries [8] and 4.1% in Asia [9]. The borderline PD itself affects approximately 0.7–2.7% of the American adults [10]. In clinics, the overall rate of PDs in psychiatric patients was reported to be about 46–58% [11], and the estimated meta-analytic PD prevalence rates of suicide attempts and self-harm were 35% and 22% respectively in hospital emergency departments [12]. On the other hand, the missed diagnosis of PDs has serious consequences, such as suicide risk, impairment in social functioning, burden of health-related suffering, and loss of productivity [13].

Additional challenges in addressing PDs involve tackling stigma and promoting early detection. The diagnosis of PDs is associated with a particular stigma, even among clinical staff [14]. These negative attitudes towards PD have adversely impacted the provision of healthcare services [15]. Therefore, there is a reluctance to diagnose PDs in younger age groups in the categorical classification systems [16]. These systems have been criticized for the lack of continuity between normal and abnormal personalities, high heterogeneity within PD categories, high PD comorbidity, high prevalence of PDs not otherwise specified, and restricted clinical ability to predict the treatment outcomes [13]. More seriously, the reluctance of PD diagnosis in younger age groups increases the risk of fatal outcomes [3].

Therefore, the precise diagnosis and early detection of PDs warrant more logical, practical systems. The dimensional systems are primarily rooted in a global severity dimension, partially encompassing personality traits inherent in PDs. These normal and disordered traits form a continuum across the lifespan [17]. Maladaptive PD traits, such as neuroticism and psychopathy, may contribute to enhanced survival, successful mating, or reproduction in humankind. Although these traits may undermine essential biological objectives, they can concurrently support others, potentially reducing the competition for finite resources [18]. The International Classification of Diseases-11 (ICD-11) has undergone a significant paradigm shift, moving away from traditional categorical descriptions of PDs to embrace dimensional perspectives [19]. Moreover, the adoption of a life-span perspective on mental disorders aims to facilitate the diagnosis of PDs in young individuals [20]. This shift not only improves the clinical utility and global applicability of diagnostic criteria, but also aids in better treatment planning, comprehensive assessment, effective communication with patients, and simplified applicability.

## Major concerns in ICD-11-based diagnoses

Regarding the diagnostic considerations, both ICD-11 and the Diagnostic and Statistical Manual of Mental Disorders-the 5th edition (DSM-5) section III (the alternative model, DSM-5-AMPD) are dimensional [21]. The ICD-11 has eliminated all traditional PDs except borderline, a departure from the International Classification of Diseases-10 (ICD-10) and has aligned more closely with the personality disorders in DSM-5-AMPD.

In the ICD-11, clinicians are initially advised to determine whether individuals meet the general diagnostic requirements of PDs, followed by evaluating the PD severity (mild, moderate, or severe) based on the impairment of self and interpersonal functions. Furthermore, a distinct delineation of five stylistic traits, namely Negative Affectivity, Detachment, Disinhibition, Dissociality and Anankastia, needs to be identified [19]. The ICD-11 trait descriptors can be applied to characterize the personality features of individuals presenting with PD or personality difficulty, thereby aiding in maintaining diagnostic continuity. Moreover, the retention of the borderline pattern aims to facilitate a smoother transition from ICD-10 to ICD-11 and to assist in identifying individuals who may be responsive to psychotherapy [22].

Preceding the introduction of ICD-11, the DSM-5-AMPD incorporated the assessment of impairment in self and interpersonal functioning, along with a distinct characterization of 25 stylistic traits organized under five domains (Negative Affectivity, Detachment, Antagonism, Disinhibition and Psychoticism) [23]. Versus the ICD-11 purely dimensional model, the DSM-5-AMPD is a hybrid dimensional-categorical model. In contrast to previous DSM versions (e.g., DSM-IV, texted revision, etc.), the DSM-5-AMPD incorporates the six individual PD types (antisocial, avoidant, borderline, narcissistic, obsessive-compulsive, and schizotypal PD), and eliminates the subclassification of “personality disorders not otherwise specified” [24] Table 1.

**Table 1:** Comparisons of DSM-5 and ICD-11 models regarding personality disorder functioning type and impairment severity*

Items	DSM-5 alternative model	ICD-11 model
Personality dysfunction	0 No impairment	None
	1 Some impairment	Personality difficulty
	2 Moderate impairment	Mild personality disorder
	3 Severe impairment	Moderate personality disorder
	4 Extreme impairment	Severe personality disorder
Trait domain	Negative affectivity	Negative affectivity
	Detachment	Detachment
	Disinhibition	Disinhibition
	Antagonism	Dissociality
	(rigid perfectionism)	Anankastia
	Psychoticism	(Schizotypal disorder)
Specific type	Six (Antisocial, Avoidant, Borderline, Narcissistic, Obsessive-Compulsive, Schizotypal) personality disorder of Trait-Specified.	Borderline pattern specifier

*Note* *, after Mulder, 2021 [24]

**Fig. 1:**
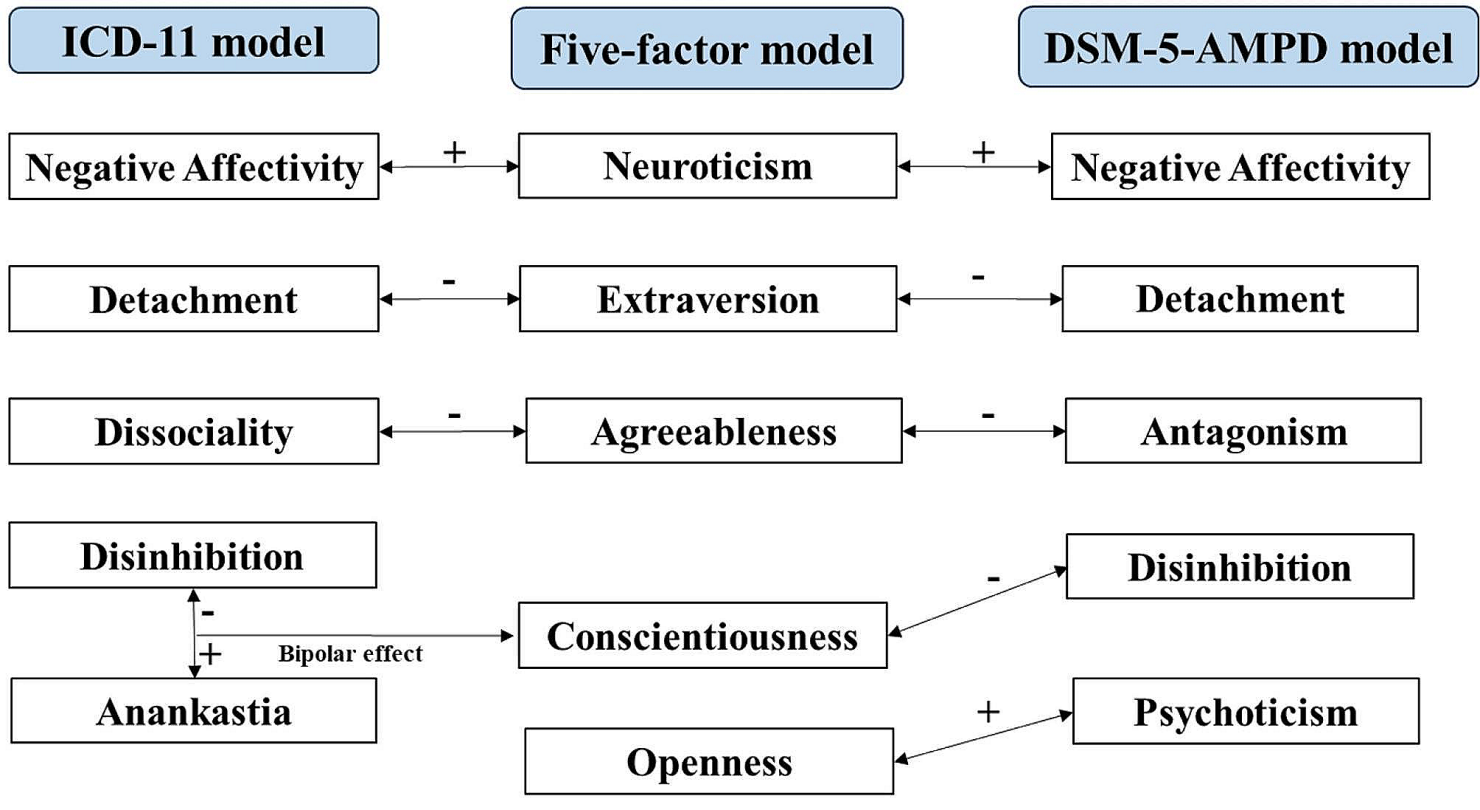
Juxtaposition of ICD-11 and DSM-5 models of personality disorder domains and the five-factor model of normal personality traits (after Strus et al., 2021 [29])

Both ICD-11 and DSM-5-AMPD dimensional models are primarily derived from psychodynamic frameworks such as Kernberg’s model [25] and the object relational models [26]. Each of 10 categorical PD types (DSM-5 domains, criterion count and binary diagnoses) can be generally predicted by the ICD-11 and DSM-5-AMPD [27] Table 2. Prior research has presented evidence of scale loadings on five personality traits [28]. Both ICD-11 and DSM-5-AMPD are connected to the big five personality traits, and in ICD-11, there exists a bipolar factor encompassing anankastia-disinhibition along the conscientiousness dimension [29, 30] (Fig. 1).

**Table 2:** Categorical to dimensional cross-walk with personality disorder domains in DSM-5 and ICD-11 models*

Personality disorder type	DSM-5 domain	ICD-11 domain
Cluster A		
Paranoid	Detachment	Detachment
	Negative Affectivity	Negative Affectivity
	Antagonism	Dissociality
Schizoid	Detachment	Detachment
	Low Negative Affectivity	Low Negative Affectivity
Schizotypal	Psychoticism	[Schizotypal Disorder]
	Detachment	Detachment
	-	(Anankastia)
Cluster B		
Antisocial	Antagonism	Dissociality
	Disinhibition	Disinhibition
	Low Negative Affectivity	Low Negative Affectivity
Borderline	Negative Affectivity	Negative Affectivity
	Disinhibition	Disinhibition
	Psychoticism	-#
Histrionic	Disinhibition	Disinhibition
	Negative Affectivity	Negative Affectivity
	Low Detachment	Low Detachment
	Antagonism	Dissociality
Narcissistic	Antagonism	Dissociality
Cluster C		
Avoidant	Negative Affectivity	Negative Affectivity
	Detachment	Detachment
	Low Antagonism	Low Dissociality
Dependent	Negative Affectivity	Negative Affectivity
	Low Antagonism	Low Dissociality
Obsessive-compulsive	-	Anankastia
	Low Disinhibition	Low Disinhibition
	Negative Affectivity	Negative Affectivity

*Note* *, after Bach et al., 2018 [27]; #, may potentially be elucidated using the ICD-11 diagnosis of complex post-traumatic stress disorder including feature of dissociation

The two diagnostic models are advantageous in differentiating PDs from other mental disorders [31]. They can also be utilized to detect the association between personality features and patients’ readmission and mortality risk [32, 33], and they are applicable to old people [34] and adolescents [35]. In addition, both ICD-11 and DSM-5-AMPD possess advantages over the categorical system in PD treatment, which is largely compatible with the Schema Therapy model [36].

### Anankastia vs. psychoticism

The components of ICD-11 and DSM-5-AMPD exhibit interrelations and align closely with specific normal personality traits [24]. However, differences exist between the two diagnostic systems, with a distinct arising in the conceptualization of the fifth dimension: anankastia in ICD-11 versus psychoticism in DSM-5. ICD-11 has eliminated the psychoticism trait due to its features different from PDs [27]. It fails to map the normal traits under the five-factor personality model, while it is incorporated with the antisocial PD [29]. Anankastia, as conceptualized in ICD-11, is closely associated with perfectionism. This trait manifests as a rigid adherence to norms and obligations, featuring emotional and behavioral constraints, such as inflexible control and perseveration. The DSM-5 trait facets corresponding to anankastia include rigid perfectionism and preservation, originating from the low Disinhibition and Negative Affective domains respectively [37]. Anankastia encompasses essential features of obsessive-compulsive PD and certain aspects of narcissistic (e.g., narcissistic perfectionism) and avoidant (e.g., risk aversion and overconcern) PDs. However, the negative associations with Disinhibition (e.g., reversed Disinhibition) do not account for these features [38]. Nevertheless, ICD-11’s anankastia exhibited satisfactory discrimination and validity across various cultures [16].

However, both ICD-11 and DSM-5-AMPD possess their own advantages when referring to anankastia and psychoticism. The ICD-11 provides a more comprehensive coverage of personality pathology compared with DSM-5-AMPD, notably due to the specificity and cohesive placement of anankastia within the overall personality structure in contrast to psychoticism [29]. While the ICD-11’s Anankastia considerably overlaps with DSM-5 obsessive-compulsive PD [39], its rigidity falls short of fully capturing the obsessive-compulsive PD construct [40]. Bach et al. have found a similar superiority in capturing obsessive-compulsive PD using ICD-11, while DSM-5-AMPD excels in capturing schizotypal PD [27]. Additionally, some case reports concentrated on the distinction advocated by the ICD-11 in the disinhibition/ anankastia personality domain, whereas the psychoticism personality domain is a DSM-5-AMPD conceptualized trait [41].

### Severity of personality dysfunction

The ICD-11 categorizes PDs into five severity levels: “No impairment, Personality Difficulty, Mild Personality Disorder, Moderate Personality Disorder, and Severe Personality Disorder.” The last three severity levels specifically pertain to clinical disorders, while the first two do not. In contrast, DSM-5-AMPD proposes five levels of impairment in personality functioning: None/ Little (0), Some (1), Moderate (2), Severe (3), and Extreme (4) Table 1. Notably, the ICD-11 not only encompasses the self and interpersonal functioning, but also includes emotional, cognitive, and behavioral manifestations. For instance, this encompasses self-harm and psychotic-like perceptions, such as disturbances in reality testing [42].

## ICD-11-related diagnostic measures

There is no structured clinical interview for the ICD-11 model, while several self-report and clinician-rating scales are existed to assess PD severity and the normal and disordered personality traits.

### Measures of severity

At present, a structured clinical interview for the ICD-11 model is unavailable. However, various self-report and clinician-rating scales exist for evaluating disorder severity, and for both normal and disordered personality traits. Long-term studies have underscored the perspective that personality pathology is not solely a criterion-defined disorder, while is also categorized by severity [7]. Notably, the severity of PDs strongly determines impairment and outcome [43]. The Personality Disorder Severity ICD-11 (PDS-ICD-11, 14 items) has been developed to evaluate self and interpersonal dysfunctions as well as emotional, cognitive, and behavioral symptoms, and psychosocial impairments. This measure uniquely captures all features of PD severity as defined in the ICD-11 model [44]. Across diverse samples, including a US community, a New Zealand mental health sample, and a Spanish mixed sample, the PDS-ICD-11 has demonstrated noticeable criterion validity and incremental validity in predicting PD impairments [44]. In a Spanish mixed sample, the PDS-ICD-11 properties are as adequate as those original instruments [45]. Its German version is acceptable in the general population, and its total score is more strongly associated with negative affectivity compared with antagonism and anankastia [46]. Further validation of PDS-ICD-11 in a community mental health sample has exhibited moderate-to-large associations with all clinician ratings, as well as more variable associations with self-report and informant-report measures [47]. Mean scores of PDS-ICD-11 were significantly different across all levels of ICD-11 PD clinician-rated diagnostic levels. In a Danish general population, practical thresholds of 12, 16, and 19 indicated mild, moderate, and severe PD [48]. An additional study of the Clinician-Rating Form of PDS-ICD-11 demonstrated that item-response theory and confirmatory factor analyses support both item functioning and uni-dimensionality [49].

The Scales of Self and Interpersonal Dysfunction (65 items) are based on the ICD-11, including six domains of self- and interpersonal dysfunctions, identity problems, relationship difficulties, and dysfunctional engagements, as well as five personality domains [50]. The psychometric properties of the Scales are excellent, as indicated by the domains and their components’ convergent and discriminant validities. However, the Scales do not cover the emotional, cognitive, and behavioral manifestations, nor the global psychosocial impairments. The scales are only preliminary and do not take the full ICD-11 severity models into account.

Other measures originally developed for the DSM-5-AMPD criterion A have recently been utilized to assess PD severity based on the ICD-11. For example, the Level of Personality Functioning Scale [21], the Level of Personality Functioning Scale - Brief Form [51], and the Level of Personality Functioning Scale-self-report [52] are the reliable measures. The Self and Interpersonal Functioning Scale is a time-efficient support for clinical decision and treatment planning under the ICD-11 framework [53]. Other measures, such as the Semi-Structured Interview for DSM-5 Personality Functioning (STiP5.1), may describe most information needed for determining PD severity based on the ICD-11 [54]. The STiP5.1 has been translated into several versions and proven to be valuable to evaluate personality functioning dimensions. Notably, it has been utilized effectively in different cultural contexts, such as the Czech [55], Estonian [56], and German [57] versions. As a specific tool for assessing personality functioning in adolescence, the Levels of Personality Functioning Questionnaire 12–18 (LoPF-Q 12–18) is available and recommended [58]. In addition, the Inventory of Personality Organization is a self-report measure which can be employed to assess three domains of personality organization [59]. Table 3

**Table 3:** Scales based ICD-11 and DSM-5 models and Kernberg’s theory of personality organization to measure personality disorder dysfunctional severity

Measurement	Item numbers	Components/ subscales	
ICD-11			
Personality Disorder Severity ICD-11 (PDS-ICD-11)	14	Identify, self-worth, self-perception, goals, interest in relationships, disagreement management, emotional control and expression, behavioral control, experience of reality during stress, harm to self, harm to others, psychosocial impairments.	
Scales of Self and Interpersonal Dysfunction (of Clark and Colleagues)	65	Low self-worth, low self-accuracy, low self-directedness, relationship difficulties, and dysfunctional, engagement	
DSM-5			
Level of Personality Functioning Scale (LPFS)	80	Identify, self- direction, empathy, intimacy	
Level of Personality Functioning Scale - Brief Form (LPFS-BF)	12	Self-functioning, interpersonal functioning, six items for each	
Level of Personality Functioning Scale-self-report (LPFS-SR)	80	Identity (21 items), Self-Direction (16 items), Empathy (23 items), Intimacy (20 items)	
Self and Interpersonal Functioning Scale (SIFS)	24	Identity, Self-Direction, Empathy, Intimacy	
Semi-structured Interview for DSM-5 Personality Functioning (STiP5.1)	28	Identity, Self-Direction, Empathy, Intimacy; 12 facets (Experience of oneself as unique, self-esteem, emotions, goals, self-reflection, understanding others, perspectives impact, connection, closeness, mutuality)	
Level of Personality Functioning Questionnaire for adolescents (LoPF-Q 12–18)	97	Identity, self-direction, empathy, and intimacy	
Kernberg’s theory of personality organization			
Inventory of Personality Organization (IPO)	57	Primitive Defenses (16 items), Reality Testing (20 items), Identity Diffusion (21 items)	

### Measures of traits

Numerous self-report instruments Table 4 [60] have been developed to measure the ICD-11 domains and subjected to the examination of their factor structure, multimethod usage, convergent and discriminant validities with other prominent, dimensional personality models (e.g., the five-factor model of normal traits), and criterion validity for important life outcomes.

The Personality Inventory for ICD-11 (PiCD) was designed to assess five maladaptive traits (Negative Affectivity, Detachment, Dissocial, Disinhibition and Anankastia) of ICD-11, involving 60 items (12 items each domain) [61]. Preliminary results have shown its adequate internal reliability, and convergent and discriminant validities [62]. PiCD has been tested in Spanish community and clinical samples [63] and in Italian adult samples [64]. Results supported the single-dimensionality for the PiCD Negative Affectivity, Detachment, and Dissocial scale items, as well as the bifactor model (confirmatory factor analysis) of PiCD Disinhibition and Anankastic items in Italian samples. All PiCD scales are significantly associated with the impairment in personality functioning [64]. An informant-report version of PiCD is the Informant-Personality Inventory for ICD-11 (IPiC), which facilitates reporting from other perspectives of a target person. In older adults, IPiC and PiCD have exhibited a moderate self-other agreement, which is associated with several important life functioning areas, and they have structural validity at the item level [65].

**Table 4:** Scales based on ICD-11 and DSM-5 models to measure personality disorder trait domain*

Measures	Number of Domains	Facets/items
ICD-11		
Personality Inventory (PiCD)	5 scales (Negative Affectivity, Dissociailty, Disinhibition, Detachment, Anankastia)	60 items, 12 items per domain
Informant Personality Inventory for ICD-11 (IPiC)	5 scales (Negative Affectivity, Dissociailty, Disinhibition, Detachment, Anankastia)	60 items, 12 items per domain
Five-factor Personality Inventory for ICD-11 (FFiCD)	5 scales (Negative Affectivity, Dissociality, Disinhibition, Detachment, Anankastia)	20 facets scales, 47 nuance scales; 121 items, 40 items for Negative Affectivity, 22 items for Dissociality, 24 items for Disinhibition, 13 items for Detachment and 22 items for Anankastia; 2–4 nuance scales for each facet (except Distrust), Unassertiveness, and Thrill-Seeking). 2–3 items per nuance.
Personality Assessment Questionnaire for ICD-11 (PAQ-11)	5 scales (Negative Affectivity, Dissociailty, Disinhibition, Detachment, Anankastia)	17 items, 3–4 items per domain
DSM-5		
Personality Inventory for DSM-5 (PID-5)	5 scales (Negative Affectivity, Detachment, Antagonism, Disinhibition, Psychoticism)	25 facets, 220 items; 4–14 items per facet

*Note* *, after Oltmanns, 2021 [60]

The Personality Assessment Questionnaire for ICD-11 personality trait domains (PAQ-11) was developed in South Korea for a rapid measurement by clinicians and researchers, exhibiting adequate convergent and discriminant validities with the five-factor model, the DSM-5 trait model and emotional difficulties [66]. When PAQ-11 is applied to a sample from a U.S. community, the findings encompass 4 out of 5 ICD-11 trait domains, namely Negative Affectivity, Detachment, Disinhibition and Anankastia. Additionally, the results prompt inquiries regarding the structural reliability of the Dissociality scale and the discriminant validity of the Disinhibition and Anankastia scales [67].

A recently proposed alternative measure to PiCD, aiming to provide a more detailed and clinically relevant depiction of personality traits, is the Five-Factor Personality Inventory for ICD-11 (FFiCD) [68]. Comprising 121 items and 20 facets, the FFiCD functions as a self-report tool, concentrating ICD-11 maladaptive traits at the facet level, with 47 short scales situated under the facets, providing a nuanced perspective. In a Spanish community sample, the FFiCD has demonstrated strong internal constructs and exhibited high correlations with other scales measuring personality functioning [69]. Other scholars have proposed that the Revised NEO Personality Inventory and the Short-form of the Zuckerman-Kuhlman-Aluja Personality Questionnaire may be complementary to PD measures or FFiCD [70, 71].

The self-report Personality Inventory for DSM-5 (PID-5), involving 220 items, 25 traits facets, and five higher traits, can also be utilized to capture ICD-11 trait domains [23]. To compute the ICD-11 domains, an algorithm based on the 16 PID-5 facet scales has been developed [37]. Both the 16 PID-5 facets in an Iranian community sample [72] and the expanded 18 PID-5 facets (including Suspiciousness and Attention Seeking) in a Canadian psychiatric sample [73] have proven to be reliable and valid to capture the pathological personality traits. In Chinese [74] and Brazilian [75] clinical-PD samples, the PID-5 has exhibited substantial deviations from normative data, suggesting its potential as an instrument for measuring pathological personality traits in psychiatric patients. Additionally, traditional assessments, such as the Minnesota Multiphasic Personality Inventory, may aid clinicians in evaluating ICD-11 personality trait dysfunctions [76].

### Measurement of borderline pattern

A specific assessment, particularly for borderline PD, the Borderline Pattern Scale, comprises 12 items and has demonstrated commendable internal consistency and convergent validity [77]. This scale evaluates the four components characterizing the borderline pattern: Affective Instability, Maladaptive Self-Functioning, Maladaptive Interpersonal-Functioning, and Maladaptive Regulation Strategies. Moreover, it exhibits satisfactory internal consistency and convergent validity, as evidenced by its correlation with four established measures: the borderline scales from the Schedule for Nonadaptive and Adaptive Personality [78], the Coolidge Axis II Inventory [79], the Wisconsin Personality Disorders Inventory [80], and the Five Factor Borderline Inventory [81].

## Diverse applications of ICD-11

An investigation conducted on a Kurdistan community and clinical sample (*N* = 3196) [82] has revealed that the ICD-11 PD trait model exhibited a better fit for the Kurdish population compared to the DSM-5-AMPD trait model. In this study, the trait domains were operationalized using empirically supported algorithms for PID-5, and Structural validity was determined through Exploratory Factor Analysis. The findings from Kurdistan demonstrated that the model fit and the expected factor structure were deemed appropriate for the ICD-11 trait model, whereas they were less adequate for DSM-5 (specifically, disinhibition did not emerge as a separate factor). Significant differences were observed in all domain and facet scores between clinical and community subsamples, with notable variations for disinhibition and dissociality/ antagonism, and comparatively less for anankastia. Hemmati et al. thus suggest that the ICD-11 trait model is more cross-culturally fitting than the DSM-5 AMPD trait model. Moreover, clinical and community samples from Brazil, Canada, China, Denmark, Korea, Spain, and the USA generally support the ICD-11 trait domains [38, 83].

Furthermore, ICD-11 has demonstrated its high acceptability and practicability in some cultures, between clinicians and patients or within patients’ families. The rationale lies in the ICD-11 approach, emphasizing traits and severity over diagnostic labels. For example, discussing a patient’s capacity to maintain a consistently positive and stable sense of self-worth, and unraveling this in terms of traits, such as self-centeredness and self-esteem, proves more straightforward than assigning a potentially stigmatizing label-like “notorious” narcissism to that patient [76]. Furthermore, findings from a survey involving 163 mental health professionals in the Zealand region of Denmark indicated that the ICD-11 PD framework is generally acceptable in terms of utility. Clinicians perceive it as comprehensive and user-friendly for describing global personality traits, irrespective of their educational background and professional experience, especially compared with the ICD-10 framework [20]. In a further clinical comparative study conducted in New Zealand (Aotearoa) regarding clinicians’ perspectives on the utility of the ICD-11 PD diagnosis, the ICD-11 system received higher ratings than the DSM-5 PD types across all six clinical metrics. These metrics include Ease of use, Communication with professionals, and Communication with patient, Describing all personality problems, Formulation of treatment planning, and Describing global personality [84].

## Treatment and social issues

### Treatment decision-making

According to the ICD-11, scholars have developed a series of patient-centered measures for PD, exhibiting the potential to improve making clinical decisions and treatment and enhance the healthcare standard for PD patients worldwide [85]. Moreover, a community team, The Boston (UK) Personality Project, has suggested that an increased awareness of personality functioning may lead to superior clinical outcomes and satisfaction for treating PDs [86].

The overall severity of PDs serves as a valuable decision-making tool for tailoring personalized medicine and determining appropriate treatment approaches and intensity. This severity level is intricately linked to various aspects, including long-term prognosis, treatment outcomes, risk of dropout, therapeutic alliance, readiness for treatment, risk of self-harm and violence, and susceptibility to dissociation and psychotic-like breaks. Additionally, it plays a role in the coherence of narrative identity, reflective functioning, and epistemic trust [87]. For example, the dialectical behavior therapy (DBT) is one of the psychotherapies for personality disorders. For mild personality disorder, therapists focus mainly on interpersonal problems and other quality-of-life issues, or the less comprehensive DBT may be considered (e.g., skills class and consultation team) with the possibility of more comprehensive treatment if problems do not improve. While for moderate and severe personality disorders, therapists focus primarily on reducing suicidal and self-harm behaviors, therapy interfering behaviors and other seriously destabilizing behaviors, thus they may apply the comprehensive DBT including the individual therapy, skills class, phone coaching, and consultation team action [87].

Psychotherapies can also be tailored based on the prominent trait domains. For patients exhibiting negative affectivity, therapies may aim to regulate anxiety, sadness, and other emotional variations. This involves helping patients to develop tolerance to distress, fostering self-compassion, enhancing mentalization, promoting acceptance of negative emotions, and acquiring stress management skills. Furthermore, research suggests that for individuals with PDs featuring blends of trait domains, treatment targeting prominent facets proves to be beneficial [76].

### Forensic and other settings

Individuals exhibiting high psychopathic traits tend to engage in more criminal activities and report a higher frequency of arrests [88]. Moreover, severe PDs are notably prevalent among those involved in homicides [89]. The ICD-11 framework facilitates the early identification of individuals at risk of developing severe PDs, enabling the implantation of timely and appropriate preventive interventions [89]. A significant legal development in the state of Victoria, Australia, underscores the consideration of PDs during sentencing for convicted offenders, highlighting the greater utility of the dimensional approach over the categorical one in forensic mental health [90]. However, challenges in forensic practice arise from potential reliability issues in assessing personality pathology, particularly when relying on self-report questionnaires [54]. Additionally, the ICD-11 diagnosis of “severe personality disorder, borderline pattern” may influence juror attitudes by introducing considerations of diminished responsibility [91].

In alternative settings, such as during the assessment before bariatric surgery, applying the dimensional ICD-11 trait models are suitable procedures for defining personality psychopathology and overall impairments of patients with obesity, which often help tailor interventions and improve surgical treatment outcomes [92].

## Developmental perspectives and implications

Mounting evidence suggests that personality undergoes changes throughout the lifespan. A meta-analysis study indicates that people increase in measures of social dominance (a facet of extraversion), conscientiousness, and emotional stability, especially in young people aged around 30 (20 to 40 years old); and the decline in trait measures of openness to experience and agreeableness are in old age [93]. In a comprehensive 30-year cohort study employing category and severity descriptions for personality diagnosis, findings revealed that 47% of patients (especially those without personality disturbances at baseline) maintained their personality statuses, 16.8% showed improvement, and 20.4% experienced a worsening to a more severe level. Notably, in patients diagnosed with DSM-III, the frequencies of Clusters A and C PDs increased from 14 to 40% over the follow-up period, underscoring the dynamic nature of PDs and their varied expressions across the lifespan [94]. Adolescence emerges as a sensitive period for the development of PDs [95], with clinical onset and peak prevalence occurring during adolescence and young adulthood [96]. An empirically epidemiological study has shown that the cumulative prevalence of PDs is about 25.7% in ages around 22 [97].

Despite this, PDs in young individuals are often underdiagnosed or face delayed diagnosis. Only 1% of young people attending a national primary care youth mental health service network receive a primary diagnosis of borderline PD or “borderline traits” [98]. This contrasts with estimates of 11–22% among outpatients and as high as 33–49% among inpatients [96]. Failing to diagnose PDs in their early stages deprives adolescents of effective treatments and increases their risk of adverse outcomes later in life [3].

Nevertheless, diagnosing PDs during adolescence remains a subject of controversy. One crucial factor is the substantial variation in the trajectories of adolescents with different personality traits as they mature [93]. Another contributing factor is the stigma associated with mental health conditions. A survey examining the 10-year stability of PDs from adolescence to young adulthood in a high-risk sample revealed a prevalence of any PD at 20.0% during baseline and 30.4% at follow-up. Significantly increased prevalence rates were observed for most PDs except for the histrionic PD [99]. For a clinical benefit, the earlier detection, diagnosing, and treatment of PDs is essential. Recognizing the potential for growth and temporary stability, ICD-11 permits the diagnosis of PDs at any age if a special trait persists over two years [19]. By incorporating a continuum of severity, ranging from none to difficulty and from mild to severe, ICD-11 moves away from specific disorders, which may contribute to a reduction of stigma associated with PDs.

A recent comprehensive overview has been conducted on instruments designed for assessing personality functioning in adolescents [100]. This review and other attempts to measure the DSM-5-AMPD styles in adolescence [101] might provide the assessment safety and decrease the related controversy. For example, the Criterion A (i.e., identity, self-direction, empathy, and intimacy) helps to assess the PD onset in adolescence, and the Criterion B provides a valuable description of continuous aspects of personality function functioning over time [102]. In general, diagnosis of PDs in adolescence facilitates the early intervention and improves both mental and physical health consequences. Notably, the structured psychological interventions have consistently demonstrated a significant improvement among young people with borderline pattern specifiers, including the reduced self-harm and suicidal ideation [103, 104]. However, the available high-quality studies regarding the effect of specialized treatments for borderline pattern in adolescence is limited, and efforts to translate adult borderline pattern psychotherapies to adolescents have exhibited minimal success [98, 105].

## Future perspectives and conclusions

The cultural feasibility, communication convenience, and treatment implications of ICD-11 have been evident in its application. However, there are several areas for potential exploration with the use of ICD-11. These include investigating the social, family, and personal relevance of reducing stigma associated with PDs, understanding the longitudinal significance of lifespan development related to PDs and their treatments, and exploring the easy applicability of PD diagnostic tools, such as the simplicity of reliable questionnaires. ICD-11 underscores that PDs may change over the lifespan, emphasizing that early intervention during adolescence can enhance overall treatment outcomes.

At present, there is no structured clinical interview specifically designed for the ICD-11 model. However, alternatives include using structured clinical interviews for DSM-5-AMPD to map personality pathology according to the ICD-11 and considering instruments, such as the STiP-5.1. While existing instruments assessing PDs according to the ICD-11 are valuable aiding diagnoses, but they are not enough to assess the personality pathology, meanwhile there is a gap in measuring treatment outcomes aligned with the ICD-11 classification. Addressing this gap may involve developing clinician-rating forms, diagnostic interviews, and treatment protocols and trials [76]. These assessments or clinical control practices hold promise for PD patients by enhancing their diagnosis, distinguishing them from other mental disorders and comorbidities, and guiding personalized treatment effectively.

In conclusion, regarding the diagnostic and treatment applications, the dimensional PD approaches in ICD-11 show promise in diagnostic and treatment applications. Continuous research is essential, especially regarding the ICD-11 implementation into clinical practice across diverse cultures, the efficacy of personalized treatment, particularly in adolescence, the development of simplified instruments supporting diagnosis, and the design of longitudinal clinical spanning different age groups (Table 5).

**Table 5:** Description contributions, practical benefits, and research incentives regarding the personality disorder classification in ICD-11

Practical needs	ICD-11 Contributions	Hints in practice	Research demands
Diagnostic accuracy	Fostering dimensional and severity descriptions	Clinical benefit	Cultural extension
	Taking developmental view	Early detection in adolescence	Longitudinal observation
Diagnostic support	Connecting with questionnaires	Easy availability (reliable and valid)	Simplicity pursuing
Therapy	Applying in adolescence	Broad lifespan	Longitudinal design
Public awareness	Understanding life-long trait changeability	Stigma reduction and treatment confidence increment	Social (family) relevance

## Data Availability

This is a review paper.

## Abbreviations

DSM-5: the fifth edition of the Diagnostic and Statistical Manual of Mental Disorders
DSM-5-AMPD: the alternative model of DSM-5
FFiCD: Five-Factor Personality Inventory for ICD-11
ICD-11: International Classification of Diseases-11
IPO: Inventory of Personality Organization
LoPF-Q 12–18: Levels of Personality Functioning Questionnaire for adolescents
LPFS: Level of Personality Functioning Scale
LPFS-BF: Level of Personality Functioning Scale - Brief Form
LPFS-SR: Level of Personality Functioning Scale-self-report
PAQ-11: Personality Assessment Questionnaire for ICD-11 personality trait domains
PD: Personality disorder
PDS-ICD-11: Personality Disorder Severity ICD-11
PiCD: Personality Inventory for ICD-11
PID-5: Self-report Personality Inventory for DSM-5
SIFS: Self and Interpersonal Functioning Scale
STiP5.1: Semi-structured Interview for DSM-5 Personality Functioning
